# Nicotine Exposure From Smoking Tobacco and Vaping Among Adolescents

**DOI:** 10.1001/jamanetworkopen.2024.62544

**Published:** 2025-03-12

**Authors:** David Hammond, Jessica L. Reid, Maciej L. Goniewicz, Ann McNeill, Richard J. O’Connor, Danielle Corsetti, Ashleigh C. Block, Leonie S. Brose, Deborah Robson

**Affiliations:** 1 School of Public Health Sciences, University of Waterloo, Waterloo, Ontario, Canada; 2 Department of Health Behavior, Roswell Park Comprehensive Cancer Center, Buffalo, New York; 3 Department of Addictions, Institute of Psychiatry, Psychology and Neuroscience, King’s College London, London, United Kingdom

## Abstract

**Importance:**

It remains unknown whether nicotine intake among youths who vape is lower, comparable, or higher than among youths who smoke.

**Objective:**

To examine potential differences in biomarkers of exposure to nicotine (1) between adolescents who smoke tobacco, vape, both vape and smoke (dual use), or do not use; (2) between adolescents in 3 countries; and (3) by nicotine content and form in the vaping product last used among adolescents who exclusively vaped.

**Design, Setting, and Participants:**

This population-based, observational cross-sectional study invited adolescents aged 16 to 19 years in Canada, England, and the US who had previously completed national surveys to participate in a biomarker study based on their vaping and smoking status. Participants completed questionnaires and self-collected urine samples between September 2019 and January 2022. Analyses were conducted in February 2023 and between January and June 2024.

**Exposures:**

Vaping, tobacco smoking, dual use, or no use in the past 7 days.

**Main Outcomes and Measures:**

Urine concentration of cotinine, trans-3′-hydroxycotinine (3OH-cotinine), and total nicotine equivalents (TNE-2; molar sum of cotinine and 3OH-cotinine), normalized for creatinine concentration.

**Results:**

Among the 364 participants (mean [SD] age, 17.6 [1.1] years; 203 females [55.8%]) who provided usable urine samples and completed questionnaires, no differences in TNE-2 concentration were observed between adolescents who exclusively vaped (n = 73; geometric mean [SD], 3.10 [16.69] nmol/mg creatinine), exclusively smoked (n = 68; geometric mean [SD], 3.78 [18.00] nmol/mg creatinine), or both vaped and smoked (n = 77; geometric mean [SD], 6.07 [19.08] nmol/mg creatinine) in the past week, adjusting for creatinine concentration, age, sex, country, and cannabis use. All vaping and/or smoking groups had higher concentrations of TNE-2 than no use (n = 146; geometric mean [SD], 0.19 [1.14] nmol/mg creatinine; *P* < .001 for all contrasts). Among adolescents who exclusively vaped (n = 73), TNE-2 concentrations were not significantly different between those who reported using products containing more than 20 mg/mL nicotine (n = 33; geometric mean [SD], 4.35 [18.25] nmol/mg creatinine) and containing 20 mg/mL nicotine or less (n = 28; geometric mean [SD], 5.13 [15.64] nmol/mg creatinine). Reported use of vaping products containing nicotine salts (n = 23) was associated with higher concentration of TNE-2 (geometric mean [SD], 10.78 [18.03] nmol/mg creatinine) than reported use of products without nicotine salts (n = 29; geometric mean [SD], 2.72 [15.42] nmol/ng creatinine; *P* = .03) or reporting “don’t know” (n = 14; geometric mean [SD], 1.55 [15.01] nmol/ng creatinine; *P* = .009). Similar patterns of exposure were observed for cotinine and 3OH-cotinine.

**Conclusions and Relevance:**

This cross-sectional study found that vaping was associated with similar nicotine exposure as smoking among adolescents. Reported use of a nicotine salt product was associated with higher nicotine exposure among those who exclusively vaped, consistent with findings from laboratory and population studies indicating greater dependence for nicotine salt e-cigarettes.

## Key Points

**Question:**

Does nicotine exposure differ between adolescents who vape, smoke, vape and smoke, or do neither and by type of vaping product?

**Findings:**

In this cross-sectional study of 364 adolescents from 3 countries, exclusively vaping in the past week was associated with similar nicotine metabolite levels compared with smoking tobacco (exclusively and both vaping and smoking). Among adolescents who exclusively vaped, those who reported using nicotine salts had higher levels of nicotine metabolites vs those who reported using nonsalt products or who did not know.

**Meaning:**

These findings suggest that nicotine intake is similar among adolescents who vape and adolescents who smoke, with potentially important implications for understanding dependence and long-term patterns of vaping.

## Teaser

This cross-sectional study compares differences in nicotine metabolite levels among adolescents in Canada, England, and the US who smoke, vape, both vape and smoke, or do not smoke or vape.

## Introduction

Nicotine is the main addictive constituent in tobacco, responsible for the reinforcing and withdrawal properties that undermine efforts to quit.^1^ Over the past decade, e-cigarettes have emerged as a popular form of nicotine delivery, particularly among adolescents and young adults. E-cigarettes are a broad class of products with 4 standard components: a mouthpiece, a reservoir for holding liquid (often referred to as *e-liquid*), a heating element, and a battery to supply power.^1^ The nicotine in e-cigarette aerosol is absorbed into the bloodstream primarily through the lungs, as is the case for conventional smoked cigarettes.^2^ Nicotine delivery via the lungs is associated with greater bioavailability and abuse liability than other routes of administration, such as oral absorption from smokeless tobacco.^3^

Nicotine exposure is assessed by measuring nicotine metabolites in biological fluids, such as urine, blood, and saliva, which are commonly referred to as biomarkers of exposure.^4^ Recent literature reviews have concluded that for most individuals, vaping results in lower nicotine exposure compared with smoking.^5^ However, nicotine exposure has also been shown to differ based on the design of e-cigarettes and the characteristics of nicotine in e-liquids.^5,6^ For example, e-liquids containing nicotine salts rather than freebase nicotine have been associated with higher levels of nicotine delivery comparable to nicotine levels from smoking.^7,8^ To date, virtually all studies of nicotine exposure from vaping have been conducted among adults who formerly smoked.^9^ There is little evidence among adolescents who vape, including those with little or no history of smoking. Nicotine exposure among individuals who have never smoked is one indicator of the potential abuse liability of vaping products among adolescents. Two studies^10,11^ from the US examined urinary metabolites of nicotine among youths who vaped. Nicotine exposure was associated with higher dependence scores, although neither study conducted formal tests to compare cotinine levels between youths who vaped, smoked, or did neither.^10,11^ Another US study found no difference in cotinine levels among youths who used e-cigarettes compared with those who smoked tobacco or who did not use either, respectively, although the small sample size of the e-cigarette group (n = 12) provided limited power.^12^ Another US study reported higher levels of nicotine metabolites among youths who exclusively vaped vs youths who did not vape (controls) but lower levels than among youths who vaped and smoked.^13^

Biomarker studies of nicotine exposure also provide a means of comparing potential differences among vaping products. Two studies conducted in adult participants examined levels of nicotine metabolites by type of vaping device: nicotine exposure was higher among those who used tank vaping devices compared with cartridges or disposable devices; however, neither study tested these differences statistically.^14,15^ Another US study also found higher nicotine biomarkers among youths who vaped pod devices (most likely containing nicotine salt e-liquids) vs nonpod devices.^9^ Understanding the potential differences in exposure from freebase and salt-based nicotine e-liquids is essential given the popularity of salt-based products among youths.^16^

The current study examined biomarkers of exposure to nicotine among adolescents in Canada, England, and the US. The study aimed to examine differences in exposure to nicotine (1) among adolescents who vape, smoke tobacco, both vape and smoke (dual use), or do not use; (2) by country among these groups; and (3) by nicotine content and form in the vaping product last used among adolescents who exclusively vaped. Specific hypotheses are outlined in eTable 1 in Supplement 1.

## Methods

### Participants

The current study was an extension of the International Tobacco Control Policy Evaluation Project (ITC) Youth Tobacco and Vaping Surveys, online surveys involving national samples of adolescents aged 16 to 19 years in Canada, England, and the US.^17,18^ All participants were provided information about the study and indicated their consent in the online survey. In addition, parental consent was obtained for participants younger than 18 years. This cross-sectional study was reviewed and received ethics clearance from a University of Waterloo Research Ethics Committee, the King’s College London Psychiatry Nursing and Midwifery Research Ethics Subcommittee, and a Roswell Park Comprehensive Cancer Center Ethics Committee. We followed the Strengthening the Reporting of Observational Studies in Epidemiology (STROBE) reporting guideline.

After completing the online ITC Youth Tobacco and Vaping Survey, respondents were recruited from commercial panels in each country (eFigure in Supplement 1). Respondents were eligible if their survey responses indicated they were in 1 of 4 groups of interest: past-week vaping only, past-week cigarette smoking only, past-week vaping and smoking (dual use), or no use (no smoking, vaping, or cannabis use in the past 30 days). Other eligibility criteria were passing a data quality check and belonging to a commercial panel allowing this additional recruitment. Initial study targets of 180 participants for each vaping and smoking status group (total n = 720) were based on power calculations for a range of biomarkers. Participants received remuneration via an Amazon.com gift card ($50 in Canada, $40 in US, and £30 in England) sent by email; in Canada, participants had a choice of an Amazon.com gift card or Interac e-Transfer payment.

### Sample Collection

Sample collection occurred between September 2019 and January 2022 (n = 17 in 2019, 257 in 2020, 89 in 2021, and 1 in 2022). A urine collection kit, which included instructions and materials required for self-collection and sample return as well as a 1-page paper-and-pencil questionnaire, was sent by courier to participants. Participants were asked to collect their first urine after waking, fill 2 sample tubes (up to 40 mL), and package them with the supplied frozen gel pack in a Styrofoam box and shipping box. The samples and questionnaires were returned by courier (priority service) to the University of Waterloo for participants in Canada or Roswell Park Comprehensive Cancer Center for participants in the US or by First Class mail (1-2 days) to the National Institute for Health and Care Research BioResource Centre Maudsley (at King’s College London) for participants in England.

Received samples were immediately placed in a −20 °C freezer for storage. According to European Union regulations, samples in England were centrifuged within 7 days of receipt to remove any cellular material. After data collection, samples in Canada and England were shipped to Roswell Park Comprehensive Cancer Center on dry ice for storage and testing. The methods for collecting and shipping the biomarkers have been previously established.^19^

### Survey Measures

On the questionnaire completed at the time of sample collection, participants self-reported when (less than 1 hour ago, 1-6 hours ago, 7-12 hours ago, 12-24 hours ago, 1-7 days ago, not at all in last 7 days) they last did each of the following: *used an e-cigarette/vaped, smoked a regular cigarette, smoked any other tobacco (cigar, cigarillo, bidi, shisha, etc); smoked cannabis/marijuana; vaped cannabis/marijuana; used smokeless tobacco (chew, pinch, snuff, snus); used nicotine replacement therapy (patches, gum, lozenges, etc) or nicotine pouches; ate grilled meat (ie, cooked over flame or charcoal, or with black grill marks); *and [item was added at wave 4 in August 2020] *were in the presence of someone smoking cigarettes or tobacco inside (home, car, etc)*. Participants were asked about the characteristics of the last e-cigarette product used, including specific brand (of the device and of the cartridge, pod, or e-liquid), flavor, whether it contained nicotine, and if so, the concentration and whether it was nicotine salt. Cigarette smoking history (never smoked, ever smoked, or smoked ≥100 cigarettes in lifetime) was ascertained from responses on the ITC Youth Tobacco and Vaping Surveys, from which the participants were recruited. The questionnaires are provided in the eAppendix in Supplement 1.

### Biomarker Testing

Urine samples were tested at the Nicotine and Tobacco Product Assessment Resource laboratory at Roswell Park Comprehensive Cancer Center. Samples were tested for 2 metabolites of nicotine (cotinine and trans-3′-hydroxycotinine [3OH-cotinine]),^20^ and concentrations were normalized for creatinine concentration to adjust for differences in hydration status on sample collection. Total nicotine equivalents (TNE-2) were calculated as the molar sum of concentrations of cotinine and 3OH-cotinine.

### Categorizing Use and Vaping Product Characteristics

Participants were classified into 1 of 4 categories based on their past 7-day (week) vaping and tobacco smoking (including smoked a regular cigarette and/or smoked any other tobacco): no use (neither vaped nor smoked), exclusive vaping (vaped but did not smoke), exclusive smoking (smoked but did not vape), or both vaping and smoking (dual use). Nicotine concentration in the last vaping product used was categorized based on responses to the nicotine presence and concentration items on the questionnaire, similar to previous studies^15^ and in accordance with the 20 mg/mL limit set in Canadian^21^ and UK^22^ regulations: 0 for no nicotine, 1 for 20 mg/mL nicotine or less, 2 for more than 20 mg/mL nicotine, and 3 for “don’t know.” Among those who reported nicotine in the last product used, use of nicotine salts was self-reported and coded 0 for no, 1 for yes, and 2 for “don’t know.”

### Statistical Analysis

Biomarker values below the assay limit of quantitation (LOQ) were imputed using the common substitution formula LOQ/√2. All biomarker values were normalized for creatinine concentration, calculated by dividing the urine’s biomarker concentration by creatinine concentration (expressed as mg/mL). Data points from participants with creatinine concentrations outside of the reference range^9^ were excluded (1 had ≤10 mg/dL; 2 had >370 mg/dL). Extreme values exceeding 3 SDs from the mean were excluded from analysis on a casewise basis. For each biomarker, the number of participants with a value above the LOQ and the geometric mean concentration were reported for each vaping and smoking status group, overall, and by country.

Analyses were preregistered on the Open Science Framework^23^ and were performed for the 3 specific study aims. For aim 1, separate linear regression models were conducted for each biomarker (using log-transformed values) to examine differences based on smoking and vaping status in the past 7 days (no use, exclusive vaping, exclusive smoking, dual use; all pairwise comparisons between groups). For aim 2 (country differences), separate models were estimated for each biomarker, including an interaction term between past-week smoking and/or vaping status and country, specifying contrasts that compared countries (all pairwise) among each smoking and/or vaping status group. For aim 3, among a subsample of adolescents reporting exclusively vaping in the past week, separate linear regression models examined differences based on 2 nicotine characteristics of the last vaping product used: self-reported nicotine concentration (no nicotine, ≤20 mg/mL nicotine, >20 mg/mL nicotine, or don’t know; all pairwise comparisons between groups) and self-reported use of nicotine salt e-liquids (no, yes, or don’t know; all pairwise comparisons between groups). All models were adjusted for creatinine concentration, age, sex, country, and cannabis use in the past 7 days (no use, exclusive vaping, exclusive smoking, or both vaping and smoking of cannabis). All comparisons in the models used 2-sided tests with a *P* < .05 significance level. Analyses were conducted in February 2023 and between January and June 2024 using IBM SPSS Statistics, version 29 (IBM).

Two sensitivity analyses were conducted for aim 1. First, the models were adjusted concurrently for any past-week use of nicotine replacement therapy (NRT), any past-week use of smokeless tobacco, and any past-week exposure to secondhand smoke (by adding variables for each to the models). Second, the models were conducted using smoking and vaping status in the past 24 hours, a more stringent measure of recent use (vs past week). As a sensitivity analysis for aim 3, beyond the preregistration, models were estimated adjusting for lifetime smoking (never, ever, or ≥100 cigarettes in lifetime).

## Results

### Sample 

Of the 934 kits mailed to participants, 371 (40.0%) were returned with a usable sample and completed questionnaire. The eFigure in Supplement 1 shows details of sample recruitment and participation. Characteristics of the 364 participants included in this analysis are shown in Table 1 by country (Canada: n = 129; England: n = 131; US: n = 104). Participants had a mean (SD) age of 17.6 (1.1) years and included 203 females (55.8%) and 161 males (44.2%). eTable 2 in Supplement 1 presents characteristics and past-week behaviors and exposures by past-week smoking and vaping categories.

**Table 1.** Participant Characteristics and Smoking or Vaping Status at Time of Sample Collection

Characteristic	Participants, No. (%)			
	Total (N = 364)	Canada (n = 129)	England (n = 131)	US (n = 104)
Age, mean (SD), y	17.6 (1.1)	17.6 (1.1)	17.6 (1.1)	17.4 (1.1)
Sex				
Male	161 (44.2)	59 (45.7)	56 (42.7)	46 (44.2)
Female	203 (55.8)	70 (54.3)	75 (57.3)	58 (55.8)
Past-week smoking and/or vaping				
No use	146 (40.1)	52 (40.3)	57 (43.5)	37 (35.6)
Exclusive vaping	73 (20.1)	35 (27.1)	14 (10.7)	24 (23.1)
Exclusive smoking^a^	68 (18.7)^b^	16 (12.4)	33 (25.2)	19 (18.3)
Dual use^a^	77 (21.2)^c^	26 (20.2)	27 (20.6)	24 (23.1)
Past-24 h smoking and/or vaping				
No use	185 (50.8)	64 (49.6)	73 (55.7)	48 (46.2)
Exclusive vaping	70 (19.2)	34 (26.4)	14 (10.7)	22 (21.2)
Exclusive smoking^a^	58 (15.9)^d^	15 (11.6)	28 (21.4)	15 (14.4)
Dual use^a^	51 (14.0)^e^	16 (12.4)	16 (12.2)	19 (18.3)
Past-week cannabis use				
No use	261 (71.7)	78 (60.5)	112 (85.5)	71 (68.3)
Exclusive vaping of cannabis	8 (2.2)	1 (0.8)	3 (2.3)	4 (3.8)
Exclusive smoking of cannabis	69 (19.0)	36 (27.9)	13 (9.9)	20 (19.2)
Vaping and smoking cannabis	25 (6.9)	13 (10.1)	3 (2.3)	9 (8.7)
Missing data	1 (0.3)	1 (0.8)	0	0
Past-week smokeless tobacco use				
No use	357 (98.1)	126 (97.7)	128 (97.7)	103 (99.0)
Used smokeless tobacco	7 (1.9)	3 (2.3)	3 (2.3)	1 (1.0)
Past-week NRT use				
No use	351 (96.4)	126 (97.7)	122 (93.1)	103 (99.0)
Used NRT	12 (3.3)	3 (2.3)	8 (6.1)	1 (1.0)
Missing data	1 (0.3)	0	1 (0.8)	0
Past-week SHS exposure^f^				
No exposure	120 (33.0)	40 (31.0)	45 (34.4)	35 (33.7)
Exposed to SHS	115 (31.6)	46 (35.7)	34 (26.0)	35 (33.7)
Missing data	129 (35.4)	43 (33.3)	52 (39.7)	34 (32.7)

Abbreviations: NRT, nicotine replacement therapy; SHS, secondhand smoke.

^a^ Exclusive smoking included cigarettes and/or other smoked tobacco (cigar, cigarillo, bidi, and shisha). Dual use included both vaping and also smoking cigarettes and/or other smoked tobacco.

^b^ Of the 68 who exclusively smoked, 53 smoked cigarettes but not other tobacco, 9 smoked both cigarettes and other tobacco, 4 smoked other tobacco but not cigarettes, and 2 smoked cigarettes but other tobacco was unknown.

^c^ Of the 77 who both smoked and vaped (dual use), 57 smoked cigarettes but not other tobacco, 16 smoked both cigarettes and other tobacco, and 4 smoked other tobacco but not cigarettes.

^d^ Of the 58 who exclusively smoked, 48 smoked cigarettes but not other tobacco, 6 smoked both cigarettes and other tobacco, 3 smoked other tobacco but not cigarettes, and 1 smoked cigarettes but other tobacco was unknown.

^e^ Of the 51 who both smoked and vaped, 40 smoked cigarettes but not other tobacco, 8 smoked both cigarettes and other tobacco, and 3 smoked other tobacco but not cigarettes.

^f^ Question added in wave 4 (2021).

### Differences in Nicotine Biomarkers by Past-Week Vaping and Smoking Status Groups (Aim 1)

**Figure 1.**
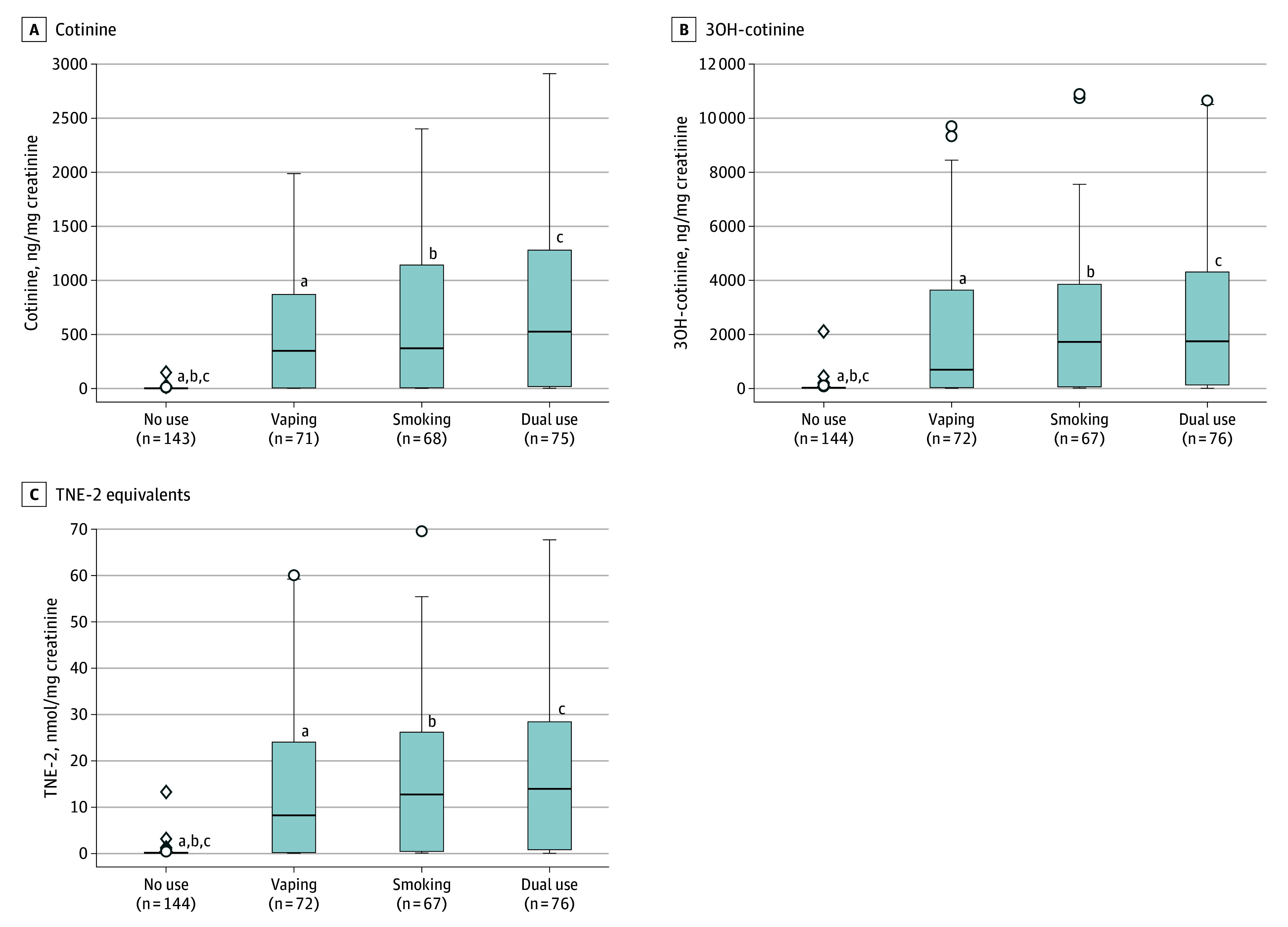
Nicotine Metabolites by Past-Week Vaping and Tobacco Smoking Status Boxplots represent median (IQR) values, whiskers represent minimum and maximum nonoutlier values for creatinine level–adjusted biomarker values within groups, and dots represent outliers. Some outliers were excluded for clarity of presentation. 3OH-cotinine indicates trans-3′-hydroxycotinine; TNE-2, total nicotine equivalents. Separate linear regression models for concentration of each biomarker (log-transformed) included testing all pairwise comparisons between past-week vaping and/or smoking status groups, adjusting for creatinine, country, age, sex, and past-week cannabis use. Significant differences between groups are noted; all other comparisons were not significantly different (see eTable 3 in Supplement 1 for model estimates and *P* values for all pairwise comparisons). ^a^Significant difference between the no use group and the vaping group (*P* < .001 for each of cotinine, 3OH-cotinine, and TNE-2, respectively). ^b^Significant difference between the no use group and the smoking group (*P* < .001 for each of cotinine, 3OH-cotinine, and TNE-2, respectively). ^c^Significant difference between the no use group and the dual use group (*P *< .001 for each of cotinine, 3OH-cotinine, and TNE-2, respectively).

Table 2 shows the number of samples with a concentration of nicotine metabolites above the LOQ within each smoking and vaping status group as well as the geometric mean concentrations of each, normalized for creatinine concentration. For example, the geometric mean [SD] concentration of TNE-2 was 3.10 (16.69) nmol/mg creatinine among those who exclusively vaped, 3.78 (18.00) nmol/mg creatinine among those who exclusively smoked, 6.07 (19.08) nmol/mg creatinine among those who vaped and smoked (dual use), and 0.19 (1.14) nmol/mg creatinine among those who did not vape or smoke (no use). Figure 1 shows box-and-whisker plots for nicotine metabolites by past-week vaping and smoking status and indicates statistically significant differences between groups, adjusting for age, sex, country, past-week cannabis use, and creatinine concentration. There were no significant differences in levels of cotinine, 3OH-cotinine, or TNE-2 among those who exclusively vaped compared with those who exclusively smoked or with dual use (eTable 3 in Supplement 1). Compared with no use, exclusive vaping was associated with higher concentrations of cotinine (β = 3.08; 95% CI, 2.47-3.69; *P* < .001), 3OH-cotinine (β = 2.40; 95% CI, 1.89-2.91; *P* < .001), and TNE-2 (β = 2.59; 95% CI, 2.07-3.11; *P* < .001), as were exclusive smoking (cotinine: β = 3.25 [95% CI, 2.64-3.86; *P* < .001]; 3OH-cotinine: β = 2.50 [95% CI, 1.98-3.01; *P* < .001]; TNE-2: β = 2.66 [95% CI, 2.13-3.19; *P* < .001]) and dual use (cotinine: β = 3.52 [95% CI, 2.89-4.16; *P* < .001]; 3OH-cotinine: β = 2.81 [95% CI, 2.29-3.34; *P* < .001]; TNE-2: β = 2.97 [95% CI, 2.43-3.51; *P* < .001]). No significant differences were observed in biomarkers of exposure between those reporting exclusive smoking and those reporting dual use (eTable 3 in Supplement 1).

**Table 2.** Presence and Concentration of Biomarkers of Nicotine Exposure in Past-Week Smoking and/or Vaping Status Groups

	Cotinine^a^		3OH-cotinine^a^		TNE-2^b^	
	No. of samples above LOQ/total No. of samples (%)	Geometric mean (SD) concentration, ng/mg creatinine^c^	No. of samples above LOQ/total No. of samples (%)	Geometric mean (SD) concentration, ng/mg creatinine^c^	No. of samples above LOQ/total No. of samples (%)	Geometric mean (SD) concentration, nmol/mg creatinine^c^
No use	13/146 (8.9)	3.25 (12.88)	8/146 (5.5)	31.55 (179.57)	NA	0.19 (1.14)
Past-week exclusive vaping	52/73 (71.2)	88.18 (557.47)	49/73 (67.1)	438.57 (2685.67)	NA	3.10 (16.69)
Past-week exclusive smoking	49/68 (72.1)	122.19 (825.89)	46/68 (67.6)	557.99 (2690.48)	NA	3.78 (18.00)
Past-week dual use	61/77 (79.2)	184.33 (811.81)	59/77 (76.6)	910.08 (2855.89)	NA	6.07 (19.08)

Abbreviations: 3OH-cotinine, trans-3′-hydroxycotinine; NA, not applicable; TNE-2, total nicotine equivalents.

^a^ Lowest limit of detection (LOD) for cotinine and 3OH-cotinine was 1.0 ng/mL. The lowest limit of quantitation (LOQ) for cotinine was 5.0 ng/mL. For 3OH-cotinine, the original lowest LOQ was 50 ng/mL for batches with samples from Canada and England but 15 ng/mL for a batch with samples from the US; thus, all values converted to the lowest LOQ of 50 ng/mL.

^b^ No estimates for presence of TNE-2 were reported, as this was calculated as the molar sum of cotinine and 3OH-cotinine, which were tested for directly.

^c^ Estimates of concentration excluded outliers (n = 4 for cotinine, n = 2 for 3OH-cotinine, and n = 2 for TNE-2) and participants with creatinine values outside of the reference range (n = 3).

### Differences in Nicotine Biomarkers by Countries (Aim 2)

Table 3 shows the geometric mean (SD) concentrations of each nicotine metabolite, normalized for creatinine concentration, in each smoking and/or vaping status group and by country. In models adjusted for age, sex, country, past-week cannabis use, and creatinine concentration, the only significant differences observed between countries within smoking and/or vaping status groups were lower concentrations of 3OH-cotinine and TNE-2 among those who exclusively smoked in Canada vs England. There was also some evidence of lower cotinine concentration among those who exclusively vaped in England vs Canada, but this comparison was not statistically significant (eTable 4 in Supplement 1).

**Table 3.** Geometric Mean Concentrations, Normalized for Creatinine Concentration, for Biomarkers of Exposure in Past-Week Smoking and/or Vaping Status Groups by Country

	No.	Geometric mean (SD) concentrations^a^		
		Cotinine, ng/mg creatinine	3OH-cotinine, ng/mg creatinine	TNE-2, nmol/mg creatinine
**Canada**				
No. of participants		127	127	127
No use	52	2.87 (4.43)	29.30 (38.11)	0.17 (0.22)
Past-week exclusive vaping	35	119.32 (590.00)	507.11 (2809.08)	3.96 (17.53)
Past-week exclusive smoking	16	108.45 (1024.42)	361.38 (2946.28)	2.64 (20.07)
Past-week dual use	26	145.42 (608.97)	842.13 (2509.87)	5.48 (16.03)
**England**				
No. of participants		127	129	129
No use	57	3.18 (4.13)	31.65 (280.25)	0.19 (1.77)
Past-week exclusive vaping	14	25.47 (291.93)	191.15 (1693.04)	1.16 (10.14)
Past-week exclusive smoking	33	136.57 (743.81)	713.51 (2561.54)	4.72 (17.06)
Past-week dual use	27	207.73 (1061.37)	928.54 (3264.00)	6.40 (22.71)
**US**				
No. of participants		103	103	103
No use	37	4.01 (24.15)	34.83 (77.09)	0.21 (0.53)
Past-week exclusive vaping	24	120.04 (578.93)	579.53 (2899.15)	3.88 (17.79)
Past-week exclusive smoking	19	111.38 (808.42)	513.01 (2810.03)	3.41 (18.76)
Past-week dual use	24	210.53 (665.09)	970.35 (2816.78)	6.42 (18.02)

Abbreviations: 3OH-cotinine, trans-3′-hydroxycotinine; TNE-2, total nicotine equivalent.

^a^ Estimates of concentration excluded outliers (n = 4 for cotinine, n = 2 for 3OH-cotinine, and n = 2 for TNE-2) and participants with creatinine values outside of the reference range (n = 3).

### Differences in Nicotine Biomarkers by Vaping Product Characteristics Among Those Who Exclusively Vaped (Aim 3)

#### Self-Reported Nicotine Concentration

**Figure 2.**
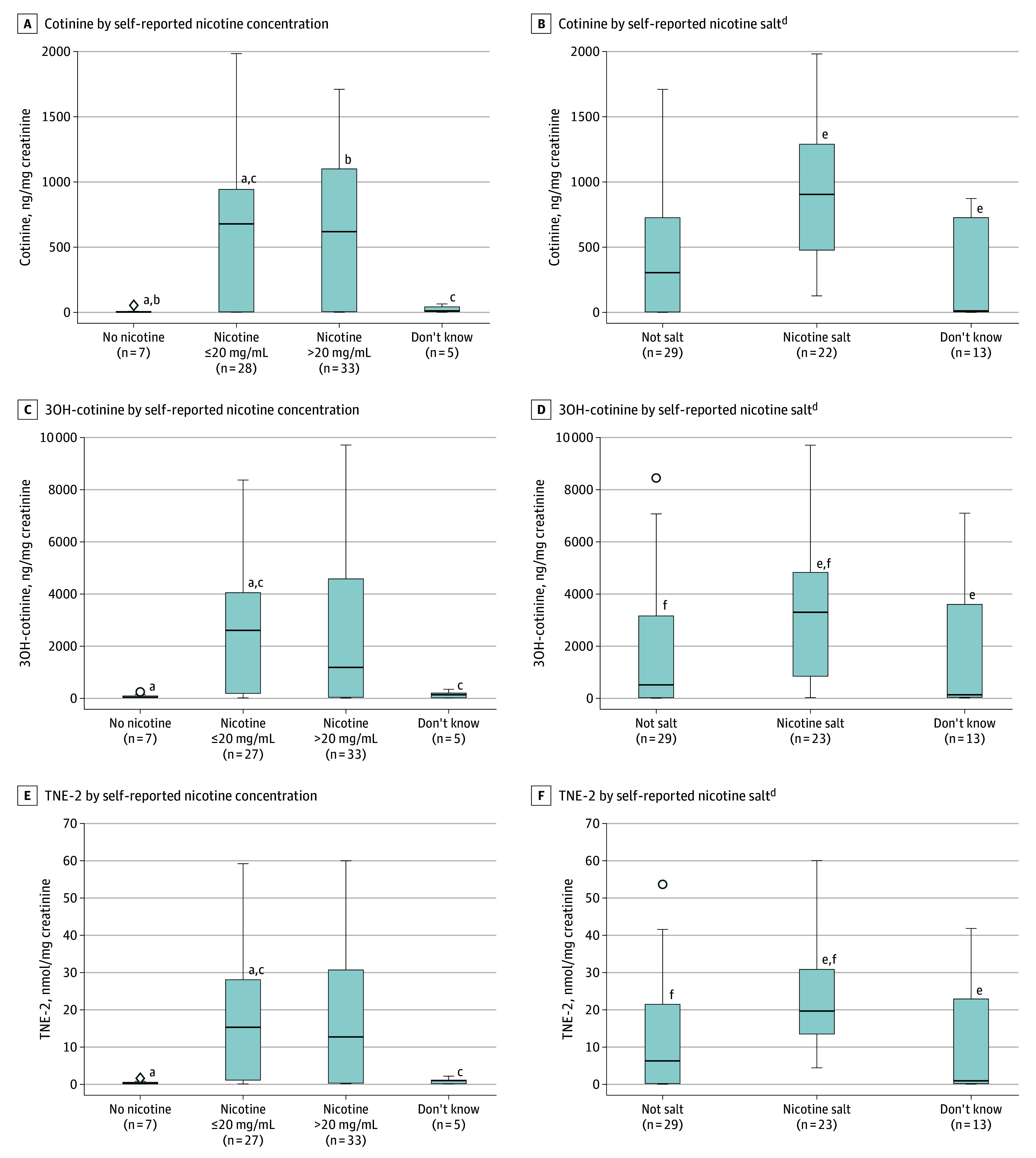
Nicotine Biomarkers by Nicotine Characteristics of Last Vaping Product Used Among Adolescents Who Reported Vaping Exclusively in the Past Week Boxplots represent median (IQR) values, whiskers represent minimum and maximum nonoutlier values for creatinine level–adjusted biomarker values within user groups, and dots represent outliers. 3OH-cotinine indicates trans-3′-hydroxycotinine; TNE-2, total nicotine equivalents. Separate linear regression models for concentration of each biomarker (log transformed) included testing all pairwise comparisons between groups, adjusting for creatinine, country, age, sex, and past-week cannabis use. Significant differences between groups are noted; all other comparisons were not significantly different (see eTable 5 and eTable 6 in Supplement 1 for model estimates and *P* values for all pairwise comparisons). ^a^Significant difference between the no nicotine group and the up to 20 mg/mL nicotine group (*P* = .001 for cotinine, *P* = .004 for 3OH-cotinine, and *P* = .002 for TNE-2, respectively). ^b^Significant difference between the no nicotine group and the more than 20 mg/mL nicotine group (*P* = .04 for cotinine). ^c^Significant difference between the up to 20 mg/mL nicotine group and the “don’t know” group (*P *= .01 for cotinine, 3OH-cotinine, and TNE-2, respectively). ^d^Excluding those reporting no nicotine in the last vaping product used. ^e^Significant difference between the nicotine salt group and the “don’t know” group (*P* = .004 for cotinine, *P *= .02 for 3OH-cotinine, and *P *= .009 for TNE-2, respectively). ^f^Significant difference between the not salt group and the nicotine salt group (*P* = .02 for 3OH-cotinine and *P* = .03 for TNE-2, respectively).

Among the 73 adolescents who exclusively vaped in the past week, 33 (45.2%) reported that the last vaping product they used contained more than 20 mg/mL nicotine, while 28 (38.4%) reported 20 mg/mL nicotine or less, 7 (9.6%) reported no nicotine, and 5 (6.8%) did not know. Adjusting for creatinine concentration, country, age, sex, and past-week cannabis use, a self-reported nicotine concentration of 20 mg/mL nicotine or less was associated with higher levels of all nicotine metabolites compared with no nicotine (eg, TNE-2: geometric mean [SD], 5.13 [15.64] vs 0.32 [0.54] nmol/mg creatinine; β = 2.60 [95% CI, 0.94-4.27; *P* = .002]) or not knowing (eg, TNE-2: geometric mean [SD], 0.52 [0.85] nmol/mg creatinine; β = 2.48 [95% CI, 0.59-4.38; *P* = .01]) but no significant differences compared with self-reported nicotine concentration higher than 20 mg/mL (TNE-2: geometric mean [SD], 4.35 [18.25] nmol/mg creatinine). The only comparison for which a concentration greater than 20 mg/mL nicotine significantly differed from other groups was for higher levels of cotinine vs no nicotine (geometric mean [SD], 143.05 [573.63] nmol/mg creatinine vs 5.75 [19.31]; β = 2.27 [95% CI, 0.10-4.43; *P* = .04) (Figure 2; eTable 5 in Supplement 1).

#### Self-Reported Nicotine Salt in e-Liquid

Among the 73 adolescents who exclusively vaped and excluding 7 who reported no nicotine in the last vaping product used, 23 (34.8%) reported that the last vaping product they used contained nicotine salt e-liquid, 29 (43.9%) reported the e-liquid was not nicotine salt, and 14 (21.2%) did not know. Self-reported use of nicotine salt was associated with higher levels of all nicotine metabolites in adjusted regression models compared with use of nonsalt products (eg, TNE-2: geometric mean [SD], 10.78 [18.03] vs 2.72 [15.42] nmol/mg creatinine; β = 1.38 [95% CI, 0.16-2.61; *P* = .03) or not knowing (eg, TNE-2: geometric mean [SD], 1.55 [15.01] nmol/mg creatinine; β = 1.94 [95% CI, 0.49-3.39; *P* = .009), although the comparison of cotinine for reporting salt (geometric mean [SD], 374.46 [582.91] ng/mg creatinine) vs nonsalt (geometric mean [SD], 85.73 [514.23] ng/mg creatinine) was not statistically significant (β = 1.37; 95% CI, –0.01 to 2.74; *P* = .051) (Figure 2; eTable 6 in Supplement 1).

### Sensitivity Analyses

For aim 1, past-week use of NRT, smokeless tobacco, and exposure to secondhand smoke were not associated with the outcomes, and there were no changes to the pattern of results; however, the model effect sizes of smoking and/or vaping status group were somewhat attenuated (eTable 7 in Supplement 1). Using smoking and vaping status in the past 24 hours (eTable 8 in Supplement 1) yielded findings similar to those for past-week use, although with slightly larger effect sizes (eTable 9 in Supplement 1). For aim 3, participants’ smoking history was not associated with any nicotine biomarker concentrations, nor did any model estimates substantially change.

## Discussion

The findings of this cross-sectional study indicate that exclusively vaping was associated with similar nicotine exposure as exclusively smoking and dual use, consistent with findings of a previous study conducted among adolescents.^9^ Levels of exposure were generally consistent across countries, with the exception of moderately higher nicotine intake for exclusive smoking in Canada vs England. The reasons for this difference are unclear and may be due to differences in the sample of smokers recruited across countries. However, the findings are generally consistent with population-based data indicating higher levels of dependence among youths who smoke in England compared with Canada and the US and with slower decreases in smoking prevalence among younger people in England in recent years.^24,25^

Previous studies of adults who vape have found lower nicotine exposure from vaping compared with smoking in general, in contrast to the current findings.^5^ However, recent trials using the current generation of e-cigarettes containing nicotine salts suggest similar levels of nicotine exposure to smoking cigarettes.^7^ A similar outcome from nicotine salts was observed in the current study: among adolescents who reported exclusively vaping, nicotine exposure was highest among those who reported using nicotine salt e-liquids. Higher nicotine intake from salt-based e-liquids may be attributable to the chemical composition and pH level of the aerosol, which reduces the bitterness and harshness of inhaling nicotine compared with freebase nicotine e-liquids.^8,26^ The current findings are consistent with results of population-based studies, in which salt-based products were associated with more frequent vaping, greater indicators of dependence, and a greater likelihood of respiratory symptoms.^9,15,27,28^ The findings are also consistent with recent laboratory studies indicating that nicotine salt formulations may have a relatively greater role in patterns of use and nicotine intake than nicotine concentration alone.^7,22^ Overall, the findings highlight the need to consider nicotine concentration and salt-based vs freebase nicotine separately. Whereas the original nicotine salt products in the US (eg, JUUL) all had high nicotine concentrations (approximately 50 mg/mL or higher), there has since been a marketwide transition to salt-based e-liquids such that even brands with lower nicotine concentration now come in salt form, especially the brands popular among adolescents.^16,29^ This use of nicotine salts even for lower concentrations is particularly common in Canada and England, which prohibit nicotine concentrations above 20 mg/mL.^20,21^ At the time the current study was conducted, the marketwide transition to nicotine salts had occurred to a greater extent in the US and Canada than in England, in which this transition appears to have occurred more recently.^16,30^ The later adoption of nicotine salt products in England likely reflects differences in national policies: England’s nicotine limit of 20 mg/mL predated the commercial release of nicotine salt e-liquids; in contrast, Canada’s limit was implemented in 2021, after salt-based products became popular among youths; no such nicotine limit exists in the US. Therefore, at the time of the current study, more participants in the US and Canada would have been vaping nicotine salt products.

### Limitations

This study is subject to general limitations associated with biomarkers of exposure. Although the method for self-collection of urine samples has previously been validated,^18^ protocol deviations may have occurred among some participants that could affect the findings. Other measures are also subject to the limitations of self-report, including the nicotine characteristics of the last vaping product used, as well as the use of cannabis and other tobacco products. Consistent with other studies, substantial proportions of adolescents who vaped reported not knowing the nicotine profile of their product.^15,31,32,33^ In many cases, product packaging makes no mention of salts, and labeling of nicotine is often obscure and inconsistent in terms of reporting by percentage or concentration (eg, 2% vs 20 mg/mL).^34^ Future studies should consider methods that do not rely on self-report, such as direct observation of images of vaping products used by participants. Categorization of smoking and vaping status based on the past week is an appropriate time frame for estimating recent exposure but does not fully account for an individual’s smoking and vaping history, which can be highly variable among adolescents. Prospective cohort studies capable of capturing changes in use over time and estimating accumulated or aggregate exposure would be particularly beneficial.

The preregistered analysis plan included a researcher-coded variable to verify the self-reported use of nicotine salt e-liquids, in which brand information reported by participants was cross-checked with product information available from manufacturers and retailers. This approach was largely practical in confirming the presence of nicotine salt in products among 20 of the 23 respondents who reported that the vaping product they used last contained nicotine salt. However, the researcher-coded approach was ineffective in verifying the form of nicotine in products reported by the remaining 43 respondents who selected no or “don’t know” to last using a nicotine salt product, due to insufficient brand information or products that are available in both salt and nonsalt versions. As a result, these analyses were excluded. Future research should perform objective verification of detailed product information during data collection.

## Conclusions

Adolescents who exclusively vaped e-cigarettes had similar nicotine exposure as those who smoked tobacco. Given the central role of nicotine in tobacco addiction, the findings suggest that the current generation of vaping products may have comparable abuse liability as traditional cigarettes. The results also indicate potentially essential differences in exposure based on product design, including the use of nicotine salt e-liquids. While salt-based e-liquids may be more appealing to adults who vape to quit smoking, they may also increase exposure among youths, potentially prolonging long-term patterns of nicotine use.

## Notes

Supplement 1. **eFigure.** Flowchart of Study Recruitment and Participation **eTable 1.** Hypotheses Associated With Each of the Three Specific Aims **eTable 2.** Participant Characteristics and Past-Week Behaviours and Exposures at Time of Sample Collection, by Past-Week Smoking and Vaping Group (n=364) **eTable 3.** Comparisons Between Past-Week Smoking/Vaping Status Groups for Biomarkers of Exposure, ng/ml **eTable 4.** Comparisons Between Countries for Creatinine-Adjusted Concentration of Nicotine Biomarkers, Within Past-Week Smoking/Vaping Status Groups **eTable 5.** Comparisons Between Self-Reported Nicotine Concentration Categories for Nicotine Biomarker Concentrations, Among Adolescents Who Exclusively Vaped in the Past Week (n=73) **eTable 6.** Comparisons Between Self-Reported Nicotine Salt Groups for Nicotine Biomarkers, Among Adolescents Who Exclusively Vaped in the Past Week, Excluding Those Who Reported Using No Nicotine in the Last Vaping Product Used (n=66) **eTable 7.** Comparisons Between Past-Week Smoking/Vaping Status Groups for Biomarkers of Exposure, ng/ml, in Sensitivity Analysis Models That Included Past-Week Smokeless Tobacco Use, Nicotine Replacement Therapy (NRT), and Secondhand Smoke (SHS) Exposure **eTable 8.** Biomarkers of Exposure Within Past-24-Hour Smoking/Vaping Status Groups, n(%) Samples With Concentration Above LOQ and Geometric Means (SD) Concentration, Normalized for mg Creatinine **eTable 9.** Comparisons Between Past-24-Hour Smoking/Vaping Status Groups for Biomarkers of Exposure, ng/ml **eAppendix.** Questionnaires

Supplement 2.
Data Sharing Statement

## References

[1] Benowitz NL. Nicotine addiction. N Engl J Med. 2010;362(24):2295-2303. doi:10.1056/NEJMra0809890 20554984 PMC2928221

[2] National Academies of Sciences, Engineering, and Medicine. Public Health Consequences of E-Cigarettes. National Academies Press; 2018. doi:10.17226/24952. 29894118

[3] Hiler M, Breland A, Spindle T, . Electronic cigarette user plasma nicotine concentration, puff topography, heart rate, and subjective effects: influence of liquid nicotine concentration and user experience. Exp Clin Psychopharmacol. 2017;25(5):380-392. doi:10.1037/pha0000140 29048187 PMC5657238

[4] WHO Study Group on Tobacco Product Regulation. Report on the scientific basis of tobacco product regulation: seventh report of a WHO study group. WHO Technical Report Series, No. 1015. October 24, 2019. Accessed June 27, 2024. https://www.who.int/publications/i/item/who-study-group-on-tobacco-product-regulation-report-on-the-scientific-basis-of-tobacco-product-regulation-seventh-report-of-a-who-study-group

[5] UK Office for Health Improvement & Disparities. Chapter 7: biomarkers of exposure. In *Nicotine Vaping in England: 2022 Evidence Update Summary*. 2022. Accessed February 6, 2023. https://www.gov.uk/government/publications/nicotine-vaping-in-england-2022-evidence-update/nicotine-vaping-in-england-2022-evidence-update-summary#chapter-7-biomarkers-of-exposure

[6] Ward AM, Yaman R, Ebbert JO. Electronic nicotine delivery system design and aerosol toxicants: a systematic review. PLoS One. 2020;15(6):e0234189. doi:10.1371/journal.pone.0234189 32497139 PMC7272070

[7] Christen SE, Hermann L, Bekka E, . Pharmacokinetics and pharmacodynamics of inhaled nicotine salt and free-base using an e-cigarette: a randomized crossover study. Nicotine Tob Res. 2024;26(10):1313-1321. doi:10.1093/ntr/ntae074 38597729 PMC11417154

[8] Cho YJ, Mehta T, Hinton A, . E-Cigarette nicotine delivery among young adults by nicotine form, concentration, and flavor: a crossover randomized clinical trial. JAMA Netw Open. 2024;7(8):e2426702. doi:10.1001/jamanetworkopen.2024.26702 39120901 PMC11316233

[9] Hartmann-Boyce J, Butler AR, Theodoulou A, . Biomarkers of potential harm in people switching from smoking tobacco to exclusive e-cigarette use, dual use or abstinence: secondary analysis of Cochrane systematic review of trials of e-cigarettes for smoking cessation. Addiction. 2023;118(3):539-545. doi:10.1111/add.16063 36208090 PMC10092879

[10] Boykan R, Messina CR, Chateau G, Eliscu A, Tolentino J, Goniewicz ML. Self-reported use of tobacco, e-cigarettes, and marijuana versus urinary biomarkers. Pediatrics. 2019;143(5):1-8. doi:10.1542/peds.2018-3531 31010908

[11] Vogel EA, Prochaska JJ, Ramo DE, Andres J, Rubinstein ML. Adolescents’ e-cigarette use: increases in frequency, dependence, and nicotine exposure over 12 months. J Adolesc Health. 2019;64(6):770-775. doi:10.1016/j.jadohealth.2019.02.019 31122507 PMC6538303

[12] Chaffee BW, Jacob P, Couch ET, Benowitz NL. Exposure to a tobacco-specific carcinogen among adolescent smokeless tobacco users in rural California, United States. Nicotine Tob Res. 2020;22(10):1764-1771. doi:10.1093/ntr/ntz147 31504879 PMC7542655

[13] Rubinstein ML, Delucchi K, Benowitz NL, Ramo DE. Adolescent exposure to toxic volatile organic chemicals from e-cigarettes. Pediatrics. 2018;141(4):1-9. doi:10.1542/peds.2017-3557 29507165 PMC5869331

[14] Rostron BL, Coleman B, Cheng YC, . Nicotine exposure by device type among adult electronic nicotine delivery system users in the Population Assessment of Tobacco and Health Study, 2015-2016. Cancer Epidemiol Biomarkers Prev. 2020;29(10):1968-1972. doi:10.1158/1055-9965.EPI-20-0317 32727724 PMC7541662

[15] Oliveri D, Liang Q, Sarkar M. Real-world evidence of differences in biomarkers of exposure to select harmful and potentially harmful constituents and biomarkers of potential harm between adult e-vapor users and adult cigarette smokers. Nicotine Tob Res. 2020;22(7):1114-1122. doi:10.1093/ntr/ntz185 31563966 PMC7291803

[16] Hammond D, Reid JL, Burkhalter R, . Trends in e-cigarette brands, devices and the nicotine profile of products used by youth in England, Canada and the USA: 2017-2019. Tob Control. 2023;32(1):19-29. doi:10.1136/tobaccocontrol-2020-056371 34099572 PMC9359003

[17] ITC Youth Tobacco and Vaping Surveys. Accessed December 12, 2024. https://davidhammond.ca/projects/e-cigarettes/itc-youth-tobacco-ecig/

[18] Hammond D, Reid JL. Trends in vaping and nicotine product use among youth in Canada, England, and the US between 2017 and 2022: evidence to inform policy. Tob Control. 2025;34(1):115-118. doi:10.1136/tc-2023-058241 37940402 PMC11610498

[19] Fix BV, O’Connor R, Hammond D, . ITC “spit and butts” pilot study: the feasibility of collecting saliva and cigarette butt samples from smokers to evaluate policy. Nicotine Tob Res. 2010;12(3):185-190. doi:10.1093/ntr/ntp191 20081040 PMC2825096

[20] Liang SH. Rapid and accurate LC-MS/MS analysis of nicotine and related compounds in urine using raptor biphenyl LC columns and MS-friendly mobile phases. 2015. Accessed January 16, 2025. https://www.researchgate.net/publication/282611632_Rapid_and_Accurate_LC-MSMS_Analysis_of_Nicotine_and_Related_Compounds_in_Urine_Using_Raptor_Biphenyl_LC_Columns_and_MS-Friendly_Mobile_Phases

[21] Government of Canada. Regulating tobacco and vaping products: vaping products regulations. August 2023. Accessed May 23, 2024. https://www.canada.ca/en/health-canada/services/smoking-tobacco/vaping/product-safety-regulation.html

[22] UK House of Commons Library. The regulation of e-cigarettes. Research briefing. January 2024. Accessed June 28, 2024. https://researchbriefings.files.parliament.uk/documents/CBP-8114/CBP-8114.pdf

[23] Hammond D, Reid J, McNeill A, . Biomarkers of exposure among youth who smoke, vape, and ‘dual use’: associations with product characteristics and potential differences across countries. Open Science Framework. Preprint posted online February 24, 2023. doi:10.17605/OSF.IO/9Z3CU

[24] Gomes MN, Reid JL, Rynard VL, . Comparison of indicators of dependence for vaping and smoking: trends between 2017 and 2022 among youth in Canada, England, and the United States. Nicotine Tob Res. 2024;26(9):1192-1200. doi:10.1093/ntr/ntae060 38531767 PMC11339172

[25] Tattan-Birch H, Brown J, Shahab L, Beard E, Jackson SE. Trends in vaping and smoking following the rise of disposable e-cigarettes: a repeat cross-sectional study in England between 2016 and 2023. Lancet Reg Health Eur. 2024;42:100924. doi:10.1016/j.lanepe.2024.100924 39070753 PMC11281926

[26] Leventhal AM, Madden DR, Peraza N, . Effect of exposure to e-cigarettes with salt vs free-base nicotine on the appeal and sensory experience of vaping: a randomized clinical trial. JAMA Netw Open. 2021;4(1):e2032757. doi:10.1001/jamanetworkopen.2020.32757 33433597 PMC7804919

[27] Adjei A, Chen B, Mantey DS, Wilkinson AV, Harrell MB. Symptoms of nicotine dependence by e-cigarette and cigarette use behavior and brand: a population-based, nationally representative cross-sectional study. Drug Alcohol Depend. 2024;255:111059. doi:10.1016/j.drugalcdep.2023.111059 38150895 PMC12239219

[28] Brose LS, Reid JL, Robson D, McNeill A, Hammond D. Associations between vaping and self-reported respiratory symptoms in young people in Canada, England and the US. BMC Med. 2024;22(1):213. doi:10.1186/s12916-024-03428-6 38807205 PMC11134717

[29] Ali FRM, Seaman EL, Crane E, Schillo B, King BA. Trends in US e-cigarette sales and prices by nicotine strength, overall and by product and flavor type, 2017-2022. Nicotine Tob Res. 2023;25(5):1052-1056. doi:10.1093/ntr/ntac284 36580384 PMC10077931

[30] Jackson SE, Brown J, Shahab L, Arnott D, Bauld L, Cox S. Nicotine strength of e-liquids used by adult vapers in Great Britain: a population survey 2016 to 2024. Addiction. Published online June 19, 2024. doi:10.1111/add.16576 38897583 PMC11813722

[31] Morean ME, Bold KW, Kong G. Adolescents’ awareness of the nicotine strength and NVP status of JUUL NVPs. Drug Alcohol Depend. 2019;204:107512. doi:10.1016/j.drugalcdep.2019.05.032 31487572 PMC6878179

[32] Pepper JK, Farrelly MC, Watson KA. Adolescents’ understanding and use of nicotine in e-cigarettes. Addict Behav. 2018;82:109-113. doi:10.1016/j.addbeh.2018.02.015 29518664

[33] Miech R, Patrick ME, O’Malley PM, Johnston LD. What are kids vaping? results from a national survey of US adolescents. Tob Control. 2017;26(4):386-391. doi:10.1136/tobaccocontrol-2016-053014 27562412 PMC5326604

[34] Rykaczewski C, Tackett AP, Klein EG, . Nicotine information disclosed online by e-cigarette brands popular with young people. Tob Prev Cessat. 2024;10:19. doi:10.18332/tpc/186953 38666216 PMC11044183

