# Omission in the Funding/Support Section

**DOI:** 10.1001/jamanetworkopen.2025.34609

**Published:** 2025-08-29

In the Original Investigation titled “Nicotine Exposure From Smoking Tobacco and Vaping Among Adolescents”^1^ published on March 12, 2025, funding for 3 authors was omitted from the Funding/Support section. The contributions of Drs McNeill, Brose, and Robson were funded by the National Institute for Health and Care Research Public Health Research program (NIHR130292). This article has been corrected.^1^
