# Supplementary material for: Nicotine Exposure From Smoking Tobacco and Vaping Among Adolescents
Source: JAMA Netw Open. 2025 Mar 12;8(3):e2462544. doi: 10.1001/jamanetworkopen.2024.62544 (PMC11904731; doi:10.1001/jamanetworkopen.2024.62544)
Supplement: Supplement 1. — eFigure. Flowchart of Study Recruitment and Participation eTable 1. Hypotheses Associated With Each of the Three Specific Aims eTable 2. Participant Characteristics and Past-Week Behaviours and Exposures at Time of Sample Collection, by Past-Week Smoking and Vaping Group (n=364) eTable 3. Comparisons Between Past-Week Smoking/Vaping Status Groups for Biomarkers of Exposure, ng/ml eTable 4. Comparisons Between Countries for Creatinine-Adjusted Concentration of Nicotine Biomarkers, Within Past-Week Smoking/Vaping Status Groups eTable 5. Comparisons Between Self-Reported Nicotine Concentration Categories for Nicotine Biomarker Concentrations, Among Adolescents Who Exclusively Vaped in the Past Week (n=73) eTable 6. Comparisons Between Self-Reported Nicotine Salt Groups for Nicotine Biomarkers, Among Adolescents Who Exclusively Vaped in the Past Week, Excluding Those Who Reported Using No Nicotine in the Last Vaping Product Used (n=66) eTable 7. Comparisons Between Past-Week Smoking/Vaping Status Groups for Biomarkers of Exposure, ng/ml, in Sensitivity Analysis Models That Included Past-Week Smokeless Tobacco Use, Nicotine Replacement Therapy (NRT), and Secondhand Smoke (SHS) Exposure eTable 8. Biomarkers of Exposure Within Past-24-Hour Smoking/Vaping Status Groups, n(%) Samples With Concentration Above LOQ and Geometric Means (SD) Concentration, Normalized for mg Creatinine eTable 9. Comparisons Between Past-24-Hour Smoking/Vaping Status Groups for Biomarkers of Exposure, ng/ml eAppendix. Questionnaires [file jamanetwopen-e2462544-s001.pdf]

Supplemental Online Content

Hammond D, Reid JL, Goniewicz ML, et al. Nicotine exposure from smoking tobacco and vaping among adolescents. *JAMA Netw Open*. Published online March 3, 2025. doi:10.1001/jamanetworkopen.2024.62544

eFigure. Flowchart of Study Recruitment and Participation

eTable 1. Hypotheses Associated With Each of the Three Specific Aims

eTable 2. Participant Characteristics and Past-Week Behaviours and Exposures at Time of Sample Collection, by Past-Week Smoking and Vaping Group

eTable 3. Comparisons Between Past-Week Smoking/Vaping Status Groups for Biomarkers of Exposure, ng/ml

eTable 4. Comparisons Between Countries for Creatinine-Adjusted Concentration of Nicotine Biomarkers, Within Past-Week Smoking/Vaping Status Groups

eTable 5. Comparisons Between Self-Reported Nicotine Concentration Categories for Nicotine Biomarker Concentrations, Among Adolescents Who Exclusively Vaped in the Past Week

eTable 6. Comparisons Between Self-Reported Nicotine Salt Groups for Nicotine Biomarkers, Among Adolescents Who Exclusively Vaped in the Past Week, Excluding Those Who Reported Using No Nicotine in the Last Vaping Product Used

eTable 7. Comparisons Between Past-Week Smoking/Vaping Status Groups for Biomarkers of Exposure, ng/ml, in Sensitivity Analysis Models That Included Past-Week Smokeless Tobacco Use, Nicotine Replacement Therapy (NRT), and Secondhand Smoke (SHS) Exposure

eTable 8. Biomarkers of Exposure Within Past-24-Hour Smoking/Vaping Status Groups, n(%) Samples With Concentration Above LOQ and Geometric Means (SD) Concentration, Normalized for mg Creatinine

eTable 9. Comparisons Between Past-24-Hour Smoking/Vaping Status Groups for Biomarkers of Exposure, ng/ml

eAppendix. Questionnaires

This supplemental material has been provided by the authors to give readers additional information about their work.

© 2025 Hammond D et al. *JAMA Network Open*

eFigure: Flowchart for participation and eligibility in biomarker study

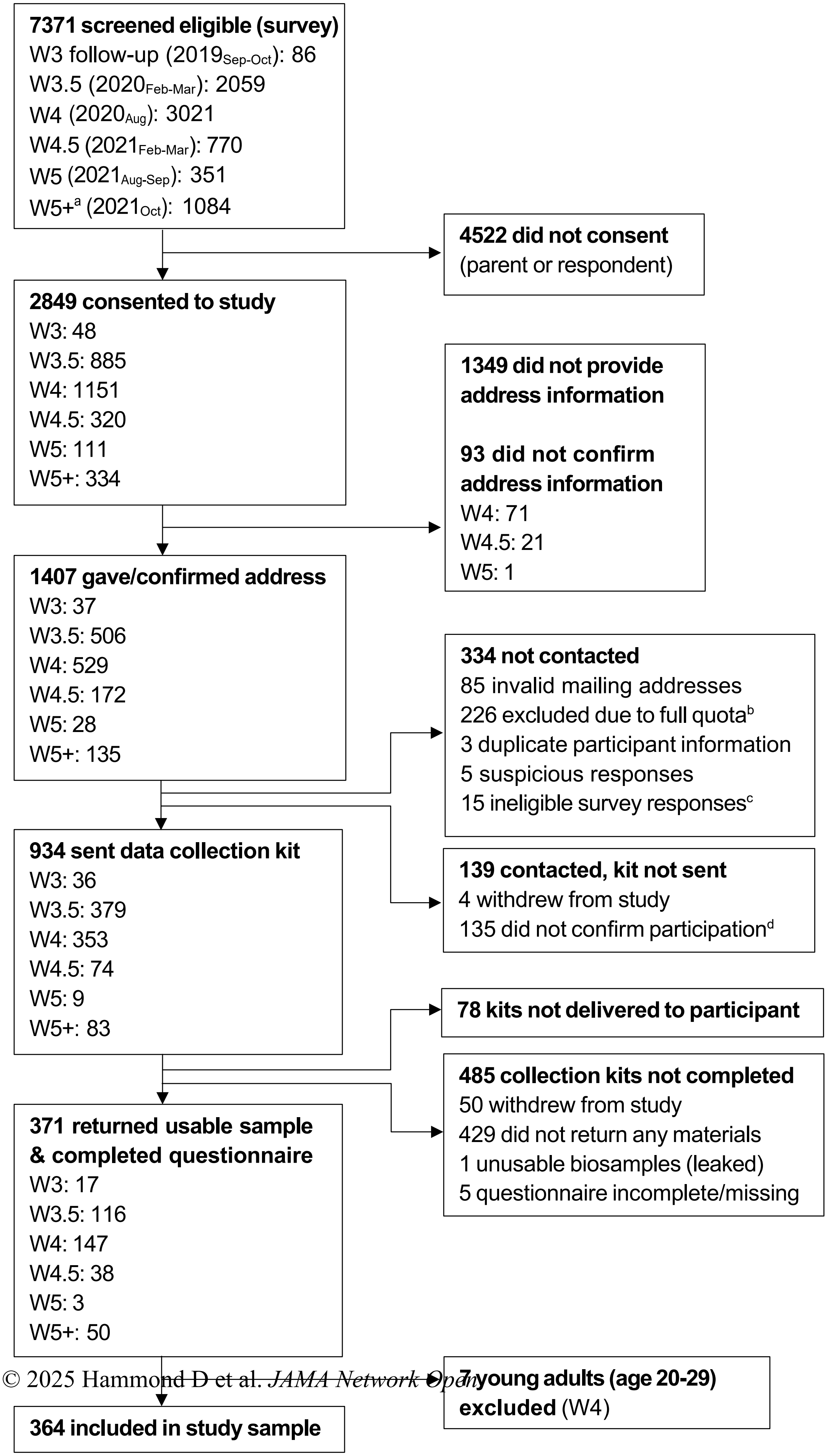

© 2025 Hammond D et al. *JAMA Network Open*

Abbreviations: W=Wave

Notes:

[a]W5+ was supplementary data collection in Canada only, conducted by Leger

[b]In W3.5 and W4, the 'non-user' groups were capped at 40 per country; no 'non-users' were recruited in W4.5, W5, W5+; no participants were contacted in England in W5

[c]In W5+, respondents who agreed to participant were asked to complete the W5 survey: 12 gave responses that indicated ineligible user status and 3 failed the data quality check question

[d]In W4.5 and W5, respondents were contacted and asked to confirm their participation before kits were sent

© 2025 Hammond D et al. *JAMA Network Open*

eTable 1: Hypotheses associated with each of the three specific aims

Specific aim	Hypotheses
1) to examine differences in exposure to nicotine among adolescents who vape, smoke tobacco, both vape and smoke, or do neither.	1. Biomarker values will vary by smoking and vaping status.
	a) Concentrations of nicotine biomarkers will be higher among those who smoke tobacco (exclusive or dual use) versus those who do not (no use and exclusive vaping), with no difference between those who exclusively smoke and those who dual use.
	b) Concentrations of nicotine biomarkers will be higher among those who vape (exclusive or dual use) than those reporting no use.
2) to examine country-level differences in exposure to nicotine.	2. Biomarker values will vary by country.
	Among those who vape (exclusive or dual use), concentrations of nicotine biomarkers will be higher among those in Canada and the US than in England.
3) among adolescents who exclusively vaped, to examine differences in exposure by nicotine content and form in the vaping product last used.	3. Among those who exclusively vaped, biomarker values will vary by product type (nicotine concentration and form in last vaping product used).
	a) Concentrations of the nicotine biomarkers will be higher among those who report using products with higher nicotine concentrations.
	b) Concentration of the nicotine biomarkers will be higher among those who report using salt-based nicotine vaping products (whether based on self-reported nicotine salt or coded based on product information).

© 2025 Hammond D et al. *JAMA Network Open*

eTable 2. Participant characteristics and past-week behaviours and exposures at time of sample collection, by past-week smoking and vaping group (n=364)

	No use n=146	Vaping n=73	Smoking[1] n=68	Dual use n=77	Total N=364
	n (%)	n (%)	n (%)	n (%)	n (%)
**Age** (mean, SD)	17.49 (1.05)	17.78 (1.08)	17.51 (1.02)	17.47 (1.18)	17.55 (1.08)
**Sex**					
Male	63 (43.2%)	25 (34.2%)	31 (45.6%)	42 (54.5%)	161 (44.2%)
Female	83 (56.8%)	48 (65.8%)	37 (54.4%	35 (45.5%)	203 (55.8%)
**Country**					
Canada	52 (35.6%)	35 (47.9%)	16 (23.5%)	26 (33.8%)	129 (35.4%)
England	57 (39.0%)	14 (19.2%)	33 (48.5%)	27 (35.1%)	131 (36.0%)
US	37 (25.3%)	24 (32.9%)	19 (27.9%)	24 (31.2%)	104 (28.6%)
**Past-week cigarette smoking**					
Did not use	146 (100%)	73 (100%)	4 (5.9%)	4 (5.2%)	227 (62.4%)
Smoked cigarettes	0 (0%)	0 (0%)	64 (94.1%)	73 (94.8%)	137 (37.6%)
**Past-week other tobacco smoking**					
Did not use	146 (100%)	72 (98.6%)	53 (77.9%)	57 (74.0%)	328 (90.9%)
Smoked other tobacco	0 (0%)	0 (0%)	13 (19.1%)	20 (26.0%)	33 (9.1%)
Missing	--	1 (1.4%)	2 (2.9%)	--	3 (0.8%)
**Past-week smokeless tobacco**					
Did not use	146 (100%)	73 (100%)	66 (97.1%)	72 (93.5%)	357 (98.1%)
Used smokeless tobacco	0 (0%)	0 (0%)	2 (2.9%)	5 (6.5%)	7 (1.9%)
**Past-week NRT use**					
Did not use	145 (99.3%)	71 (97.3%)	65 (95.6%)	70 (909%)	351 (96.4%)
Used NRT	1 (0.7%)	2 (2.7%)	3 (4.4%)	6 (7.8%)	12 (3.3%)
Missing	--	--	--	1 (1.3%)	1 (0.3%)
**Past-week SHS exposure**[2]					
No exposure	61 (41.8%)	31 (42.5%)	14 (20.6%)	14 (18.2%)	120 (33.0%)
Exposed to SHS	24 (16.4%)	23 (31.5%)	27 (39.7%)	41 (53.2%)	115 (31.6%)
Missing	61 (41.8%)	19 (26.0%)	27 (39.7%)	22 (28.6%)	129 (35.4%)
**Past-week cannabis smoking**					
Did not use	140 (95.9%)	44 (60.3%)	48 (70.6%)	35 (45.5%)	267 (73.4%)
Smoked cannabis	6 (4.1%)	29 (39.7%)	20 (29.4%)	39 (50.6%)	94 (35.8%)
Missing	--	--	--	3 (3.9%)	3 (0.8%)
**Past-week cannabis vaping**					
Did not use	144 (98.6%)	61 (83.6%)	65 (95.6%)	61 (79.2%)	331 (90.9%)
Vaped cannabis	2 (1.4%)	12 (16.4%)	3 (4.4%)	16 (20.8%)	33 (9.1%)

[1]Includes cigarettes and other smoked tobacco (cigar, cigarillo, bidi, shisha, etc.)
[2]Question about SHS added in Wave 4 (2021)
NRT=nicotine replacement therapy
SHS=secondhand smoke

© 2025 Hammond D et al. *JAMA Network Open*

eTable 3: Comparisons between past-week smoking/vaping status groups[1] for biomarkers of exposure, ng/ml

	Smoking/vaping status	Vaped vs No use	Smoked vs No use	Dual use vs No use	Vaped v Smoked	Vaped v Dual use	Smoked v Dual use
	**Model effect**	**B [95%CI] (p value) for comparison**					
**Cotinine** (n=356)	**Wald X^2=185.26 (p<0.001)**	**3.08 [2.47,3.69] (p<0.001)**	**3.25 [2.64,3.86] (p<0.001)**	**3.52 [2.89,4.16] (p<0.001)**	-0.17 [-0.87,0.53] (p=0.64)	-0.45 [-1.12,0.23] (p=0.20)	-0.28 [-0.96,0.40] (p=0.43)
**3OH-Cotinine** (n=358)	**Wald X^2=162.83 (p<0.001)**	**2.40 [1.89,2.91] (p<0.001)**	**2.50 [1.98,3.01] (p<0.001)**	**2.81 [2.29,3.34] (p<0.001)**	-0.10 [-0.69,0.49] (p=0.75)	-0.41 [-0.98,0.15] (p=0.15)	-0.32 [-0.89,0.26] (p=0.28)
**TNE-2[2]** (n=358)	**Wald X^2=176.33 (p<0.001)**	**2.59 [2.07,3.11] (p<0.001)**	**2.66 [2.13,3.19] (p<0.001)**	**2.97 [2.43,3.51] (p<0.001)**	-0.07 [-0.67,0.54] (p=0.82)	-0.38 [-0.96,0.20] (p=0.20)	-0.31 [-0.90,0.28] (p=0.30)

[1]From separate linear regression models for each biomarker (using log transformed values) adjusted for creatinine, age, sex, country, and cannabis use in the past 7 days (no use, exclusive vaping, exclusive smoking, both vaping and smoking)
[2]nmol/mg

© 2025 Hammond D et al. *JAMA Network Open*

eTable 4: Comparisons between countries for creatinine-adjusted concentration of nicotine biomarkers, within past-week smoking/vaping status groups[1]

		Canada vs England	Canada vs US	England vs US
		**B [95%CI] (p value) for comparison**		
**Cotinine** (n=356) ng/mg creatinine	**No use**	-0.06 [-0.84,0.72] (p=0.88)	-0.22 [-1.08,0.64] (p=0.61)	-0.16 [-1.01,0.69] (p=0.71)
	**Vaping**	1.25 [-0.04,2.54] (p=0.056)	0.06 [-1.04,1.16] (p=0.92)	-1.20 [-2.58,0.19] (p=0.09)
	**Smoking**	-0.82 [-2.07,0.43] (p=0.20)	-0.28 [-1.64,1.09] (p=0.69)	0.54 [-0.62,1.70] (p=0.36)
	**Dual use**	-0.77 [-1.93,0.37] (p=0.18)	-0.96 [-2.13,0.22] (p=0.11)	-0.18 [-1.33,0.97] (p=0.76)
**3OH-Cotinine** (n=358) ng/mg creatinine	**No use**	-0.05 [-0.70,0.60] (p=0.88)	-0.90 [-0.81,0.63] (p=0.81)	-0.04 [-0.75,0.68] (p=0.91)
	**Vaping**	0.59 [-0.49,1.68] (p=0.28)	-0.14 [-1.07,0.78] (p=0.76)	-0.74 [-1.91,0.43] (p=0.21)
	**Smoking**	**-1.24 [-2.32,-0.17] (p=0.024)**	-0.60 [-1.77-0.58] (p=0.32)	0.65 [-0.33,1.62] (p=0.19)
	**Dual use**	-0.47 [-1.43,0.49] (p=0.34)	-0.70 [-1.69,0.28] (p=0.16)	-0.24 [-1.20,0.72] (p=0.63)
**TNE-2** (n=358) nmol/mg creatinine	**No use**	-0.06 [-0.73,0.61] (p=0.86)	-0.11 [-0.85,0.63] (p=0.77)	-0.05 [-0.78,0.68] (p=0.89)
	**Vaping**	0.87 [-0.24,1.98] (p=0.12)	0.01 [-0.93,0.95] (p=0.98)	-0.86 [-2.05,0.33] (p=0.16)
	**Smoking**	**-1.12 [0.02,2.21] (p=0.046)**	-0.48 [-1.68,0.72] (p=0.43)	0.64 [-0.36,1.64] (p=0.21)
	**Dual use**	-0.53 [-1.51,0.45] (p=0.29)	-0.72 [-1.73,0.29] (p=0.16)	-0.19 [-1.17,0.79] (p=0.70)

[1]Simple effects of country from the interaction term for country by past-week smoking/vaping status from separate linear regression models for each biomarker (using log transformed values), adjusting for creatinine, age, sex, country, cannabis use in the past 7 days (no use, exclusive vaping, exclusive smoking, both vaping and smoking); country comparisons are within each smoking/vaping status group

Note: Estimates of concentration exclude outliers (n=4 for cotinine; n=2 for 3OH-cotinine; n=2 for TNE-2) and participants with creatinine values outside of the reference range (n=3).

© 2025 Hammond D et al. *JAMA Network Open*

eTable 5: Comparisons between self-reported nicotine concentration categories for nicotine biomarker concentrations[1], among adolescents who exclusively vaped in the past week (n=73)

	**Self-reported nicotine concentration**		**>20mg** (n=33) **v ≤20mg** (n=26/27)	**>20mg v no nicotine** (n=7)	**>20mg** v **Don't know** (n=5)	**≤20mg v no nicotine**	**≤20mg v Don't know**	**Don't know v no nicotine**
	**Geometric means (SD)**	**Model effect**	**B [95% CI] (p value) for comparison**					
**Cotinine** (n=71) ng/mg creatinine	No nicotine: 5.75 (19.31)<br>≤20mg: 144.62 (549.33)<br>>20mg: 143.05 (573.63)<br>Don't know: 12.64 (27.07)	**Wald X^2=14.74 (p=0.002)**	0.83 [-0.60,2.25] (p=0.26)	**2.27 [0.10,4.43] (p=0.040)**	1.85 [-0.27,3.97] (p=0.087)	**3.09 [1.22,4.96] (p=0.001)**	**2.68 [0.56,4.79] (p=0.013)**	0.41 [-2.22,3.04] (p=0.76)
**3OH-Cotinine** (n=72) ng/mg creatinine	No nicotine: 54.51 (83.82)<br>≤20mg: 752.69 (2456.68)<br>>20mg: 563.73 (3026.71)<br>Don't know: 83.86 (137.24)	**Wald X^2=12.96 (p=0.005)**	1.00 [-0.28,2.27] (p=0.13)	1.46 [-.050,3.42] (p=0.14)	1.43 [-.49,3.35] (p=0.15)	**2.46 [0.77,4.14] (p=0.004)**	**2.43 [0.51,4.34] (p=0.013)**	0.03 [-2.35,2.41] (p=0.98)
**TNE-2** (n=72) nmol/mg creatinine	No nicotine: 0.32 (0.54)<br>≤20mg: 5.13 (15.64)<br>>20mg: 4.35 (18.25)<br>Don't know: 0.52 (0.85)	**Wald X^2=14.28 (p=0.003)**	0.81 [-0.45,2.07] (p=0.21)	1.80 [-0.14,3.73] (p=0.069)	1.67 [-0.22,3.57] (p=0.084)	**2.60 [0.94,4.27] (p=0.002)**	**2.48 [0.59,4.38] (p=0.010)**	0.12 [-2.23,2.47] (p=0.92)

[1]From separate linear regression models for each biomarker (using log transformed values) adjusted for creatinine, age, sex, country, and cannabis use in the past 7 days (no use, exclusive vaping, exclusive smoking, both vaping and smoking)

© 2025 Hammond D et al. *JAMA Network Open*

eTable 6: Comparisons between self-reported nicotine salt groups for nicotine biomarkers[1], among adolescents who exclusively vaped in the past week, excluding those who reported using no nicotine in the last vaping product used (n=66)

	**Self-reported nicotine salt**		**Salt** (n=22/23) **v no salt** (n=29)	**Salt v don't know** (n=13)	**Don't know v no salt**
	**Geometric means (SD)**	**Model effect**	**B [95%CI] (p value) for comparison**		
**Cotinine** (n=64) ng/mg creatinine	No: 85.73 (514.23)<br>Yes: 374.46 (582.91)<br>Don't know: 35.35 (399.00)	**Wald X^2=8.79 (p=0.012)**	1.37 [-0.01,2.74] (p=0.051)	**2.36 [0.74,3.98] (p=0.004)**	-0.99 [-2.61,0.63] (p=0.23)
**3OH-Cotinine** (n=65) ng/mg creatinine	No: 356.27 (2546.40)<br>Yes: 1459.81 (2945.25)<br>Don't know: 255.28 (2466.36)	**Wald X^2=7.86 (p=0.020)**	**1.52 [0.30,2.79] (p=0.015)**	**1.71 [0.24,3.18] (p=0.023)**	-0.16 [-1.65,1.32] (p=0.83)
**TNE-2** (n=65) nmol/mg creatinine	No: 2.72 (15.42)<br>Yes: 10.78 (10.78)<br>Don't know: 1.55 (15.01)	**Wald X^2=8.43 (p=0.015)**	**1.38 [0.16,2.61] (p=0.027)**	**1.94 [0.49,3.39] (p=0.009)**	-0.56 [-2.02,0.91] (p=0.46)

[1]From separate linear regression models for each biomarker (using log transformed values) adjusted for creatinine, age, sex, country, and cannabis use in the past 7 days (no use, exclusive vaping, exclusive smoking, both vaping and smoking)

© 2025 Hammond D et al. *JAMA Network Open*

eTable 7: Comparisons between past-week smoking/vaping status groups[1] for biomarkers of exposure, ng/ml, in sensitivity analysis models that included past-week smokeless tobacco use, nicotine replacement therapy (NRT), and secondhand smoke (SHS) exposure

	Smoking/ vaping status	Past-week smokeless	Past-week NRT	Past-week SHS	Vaped vs No use	Smoked vs No use	Dual use vs No use	Vaped v Smoked	Vaped v Dual use	Smoked v Dual use
	**Model effect**				**B [95%CI] (p value) for comparison**					
**Cotinine** (n=356)	**Wald X^2=88.3 (p<0.001)**	Wald X^2=2.9 (p=0.089)	Wald X^2=0.001 (p=0.98)	Wald X^2=0.00 (p=0.99)	**3.01 [2.23,3.79] (p<0.001)**	**2.76 [1.91,3.60] (p<0.001)**	**3.40 [2.57,4.24] (p<0.001)**	0.25 [-0.65,1.16] (p=0.58)	-0.40 [-1.24,0.45] (p=0.36)	-0.65 [-1.52,0.22] (p=0.14)
**3OH-Cotinine** (n=358)	**Wald X^2=81.4 (p<0.001)**	Wald X^2=2.8 (p=0.096)	Wald X^2=0.6 (p=0.43)	Wald X^2=0.02 (p=0.88)	**2.45 [1.80,3.10] (p<0.001)**	**2.05 [1.34,2.75] (p<0.001)**	**2.76 [2.06,3.45] (p<0.001)**	0.41 [-0.35,1.16] (p=0.29)	-0.30 [-1.01,0.41] (p=0.40)	-0.71 [-1.44,0.02] (p=0.06)
**TNE-2[2]** (n=358)	**Wald X^2=84.7 (p<0.001)**	Wald X^2=2.9 (p=0.089)	Wald X^2=0.5 (p=0.49)	Wald X^2=0.03 (p=0.86)	**2.57 [1.90,3.24] (p<0.001)**	**2.19 [1.46,2.91] (p<0.001)**	**2.88 [2.16,3.60] (p<0.001)**	0.39 [-0.39,1.16] (p=0.33)	-0.31 [-1.04,0.42] (p=0.41)	-0.69 [-1.44,0.06] (p=0.07)

[1]From separate linear regression models for each biomarker (using log transformed values) adjusted for creatinine, age, sex, country, cannabis use in the past 7 days (no use, exclusive vaping, exclusive smoking, both vaping and smoking), any smokeless tobacco use in the past 7 days, any NRT use in the past 7 days, any SHS exposure in the past 7 days
[2]nmol/mg

© 2025 Hammond D et al. *JAMA Network Open*

eTable 8: Biomarkers of exposure within past-24-hour smoking/vaping status groups, n(%) samples with concentration above LOQ and geometric means (SD) concentration, normalized for mg creatinine

	Cotinine	3OH-Cotinine	Total nicotine (TNE-2)
**PRESENCE** [n present/ total (%)]			
**No use**	26/185 (14.1%)	16/185 (8.6%)	n/a[2]
**Past-24-hour vaping**	12/70 (82.9%)	56/70 (80.0%)	n/a[2]
**Past-24-hour smoking**	48/58 (82.8%)	48/58 (82.8%)	n/a[2]
**Dual use (past-24h vaping and smoking)**	43/51 (84.3%)	42/51 (82.4%)	n/a[2]
**CONCENTRATION[1]** [geometric mean (SD)]	ng/mg creatinine	ng/mg creatinine	nmol/mg creatinine
**No use**	4.01 (174.12)	36.41 (519.62)	0.22 (3.68)
**Past-24-hour vaping**	180.77 (673.36)	858.78 (2764.19)	6.19 (17.49)
**Past-24-hour smoking**	266.52 (854.94)	1098.94 (2769.62)	7.62 (18.49)
**Dual use (past-24h vaping and smoking)**	270.10 (738.77)	1168.73 (2927.59)	7.97 (19.04)

*>95% of samples had levels below the level of quantification

[1]**Note**: Estimates of concentration exclude outliers (n=4 for cotinine; n=2 for 3OH-cotinine; n=2 for TNE-2) and participants with creatinine values outside of the reference range (n=3).

[2]No estimates for presence of TNE-2 are reported, as this was calculated as the molar sum of cotinine and 3OH-cotinine, which were tested for directly.

© 2025 Hammond D et al. *JAMA Network Open*

eTable 9: Comparisons between past-24-hour smoking/vaping status groups[1] for biomarkers of exposure, ng/ml

	Smoking/vaping status	Vaped vs No use	Smoked vs No use	Dual use vs No use	Vaped v Smoked	Vaped v Dual use	Smoked v Dual use
	**Model effect**	**B [95%CI] (p value) for comparison**					
**Cotinine** (n=356)	**Wald X^2=342.3 (p<0.001)**	**3.76 [3.23,4.29] (p<0.001)**	**3.93 [3.39,4.48] (p<0.001)**	**3.86 [3.24,4.48] (p<0.001)**	-0.17 [-0.82,0.47] (p=0.60)	-0.10 [-0.77,0.57] (p=0.77)	0.07 [-0.62,0.76] (p=0.84)
**3OH-Cotinine** (n=358)	**Wald X^2=316.7 (p<0.001)**	**3.08 [2.64,3.53] (p<0.001)**	**3.13 [2.68,3.59] (p<0.001)**	**3.02 [2.50,3.54] (p<0.001)**	-0.05 [-0.59,0.49] (p=0.86)	0.06 [-0.50,0.62] (p=0.82)	0.11 [-0.47,0.69] (p=0.71)
**TNE-2[2]** (n=358)	**Wald X^2=341.7 (p<0.001)**	**3.31 [2.86,3.76] (p<0.001)**	**3.26 [2.80,3.72] (p<0.001)**	**3.17 [2.64,3.69] (p<0.001)**	0.05 [-0.50,0.60] (p=0.85)	0.14 [-0.42,0.71] (p=0.62)	0.09 [-0.50,0.68] (p=0.76)

[1]From separate linear regression models for each biomarker (using log transformed values) adjusted for creatinine, age, sex, country, and cannabis use in the past 7 days (no use, exclusive vaping, exclusive smoking, both vaping and smoking)
[2]nmol/ml

© 2025 Hammond D et al. *JAMA Network Open*

eAppendix. Questionnaires

IMPORTANT: PLEASE FILL THIS OUT IMMEDIATELY AFTER COLLECTING YOUR URINE SAMPLE.

For the urine sample quality control purposes, please answer these essential questions:

DATE OF COLLECTION: _______________ TIME OF COLLECTION _______________am / pm

When was the LAST time you did the following…
Please circle one response per product (row).

**Used an e-cigarette/vaped**	Less than 1 hour ago	1-6 hours ago	7-12 hours ago	12-24 hours ago	1-7 days ago	Not at all in last 7 days
**Smoked a regular cigarette**	Less than 1 hour ago	1-6 hours ago	7-12 hours ago	12-24 hours ago	1-7 days ago	Not at all in last 7 days
**Smoked any other tobacco** (cigar, cigarillo, bidi, shisha, etc.)	Less than 1 hour ago	1-6 hours ago	7-12 hours ago	12-24 hours ago	1-7 days ago	Not at all in last 7 days
**Smoked cannabis/ marijuana**	Less than 1 hour ago	1-6 hours ago	7-12 hours ago	12-24 hours ago	1-7 days ago	Not at all in last 7 days
**Vaped cannabis/ marijuana**	Less than 1 hour ago	1-6 hours ago	7-12 hours ago	12-24 hours ago	1-7 days ago	Not at all in last 7 days
**Used smokeless tobacco** (chew, pinch, snuff, snus)	Less than 1 hour ago	1-6 hours ago	7-12 hours ago	12-24 hours ago	1-7 days ago	Not at all in last 7 days
**Used nicotine replacement therapy** (patches, gum, lozenges, etc.) **or nicotine pouches**	Less than 1 hour ago	1-6 hours ago	7-12 hours ago	12-24 hours ago	1-7 days ago	Not at all in last 7 days
**Ate grilled meat**	Less than 1 hour ago	1-6 hours ago	7-12 hours ago	12-24 hours ago	1-7 days ago	Not at all in last 7 days

Please tell us more about the LAST e-cigarette you used:

1. What brand was the device?
   (*please be as specific as possible*) ____
2. What brand was the cartridge, pod or e-liquid?
   (*please be as specific as possible*) ____
3. a) Did it contain nicotine? (*circle one*)

   Yes
   No
   Don't know

   b) **If Yes**: Was it nicotine salt? (*circle one*)

   Yes
   No
   Don't know

PLEASE ENSURE YOU INCLUDE THIS FORM WITH YOUR URINE SAMPLES IN THE ENVELOPE PROVIDED.

WAVE 3.5 – UPDATES

IMPORTANT: PLEASE FILL THIS OUT IMMEDIATELY AFTER COLLECTING YOUR URINE SAMPLE.

Note: We will NOT test for any illegal substances. Questions about cannabis are for quality control of samples.

DATE OF COLLECTION: _______________ TIME OF COLLECTION: _______________am / pm

When was the LAST time you did the following…

Please circle one response per product (row). If you have never done the following, select 'Not at all in last 7 days'.

**Used an e-cigarette/vaped**	Less than 1 hour ago	1-6 hours ago	7-12 hours ago	12-24 hours ago	1-7 days ago	Not at all in last 7 days
**Smoked a regular cigarette**	Less than 1 hour ago	1-6 hours ago	7-12 hours ago	12-24 hours ago	1-7 days ago	Not at all in last 7 days
**Smoked any other tobacco** (cigar, cigarillo, bidi, shisha, etc.)	Less than 1 hour ago	1-6 hours ago	7-12 hours ago	12-24 hours ago	1-7 days ago	Not at all in last 7 days
**Smoked cannabis/ marijuana**	Less than 1 hour ago	1-6 hours ago	7-12 hours ago	12-24 hours ago	1-7 days ago	Not at all in last 7 days
**Vaped cannabis/ marijuana**	Less than 1 hour ago	1-6 hours ago	7-12 hours ago	12-24 hours ago	1-7 days ago	Not at all in last 7 days
**Used smokeless tobacco** (chew, pinch, snuff, snus)	Less than 1 hour ago	1-6 hours ago	7-12 hours ago	12-24 hours ago	1-7 days ago	Not at all in last 7 days
**Used nicotine replacement therapy** (patches, gum, lozenges, etc.) **or nicotine pouches**	Less than 1 hour ago	1-6 hours ago	7-12 hours ago	12-24 hours ago	1-7 days ago	Not at all in last 7 days
**Ate grilled meat** (i.e, cooked over flame or charcoal, or with black grill marks)	Less than 1 hour ago	1-6 hours ago	7-12 hours ago	12-24 hours ago	1-7 days ago	Not at all in last 7 days

Please tell us more about the LAST e-cigarette you used *(if applicable)*:

1. What brand was the device? (*please be as specific as possible*) ____________________
2. What brand was the cartridge, pod or e-liquid? (*please be as specific as possible*) ____________________
3. What flavour was the cartridge, pod or e-liquid? ____________________
4. a) Did it contain nicotine? (*circle one*)

   Yes →
   No
   Don't know

   b) What concentration of nicotine was it? (*answer in mg/ml OR %*)

   _________mg/ml **OR** _______% nicotine

   Don't know

   c) Was it nicotine salt? (*circle one*)

   Yes
   No
   Don't know

PLEASE INCLUDE THIS FORM WITH YOUR URINE SAMPLES IN THE ENVELOPE PROVIDED.

WAVE 4 – UPDATES

IMPORTANT: PLEASE FILL THIS OUT IMMEDIATELY AFTER COLLECTING YOUR URINE SAMPLE.

Note: We will NOT test for any illegal substances. Questions about cannabis are for quality control of samples.

DATE OF COLLECTION: _______________ TIME OF COLLECTION: _______________am / pm

When was the LAST time you did the following…

Please circle one response per row. If you have never done the following, select 'Not at all in last 7 days'.

**Used an e-cigarette/vaped**	Less than 1 hour ago	1-6 hours ago	7-12 hours ago	12-24 hours ago	1-7 days ago	Not at all in last 7 days
**Smoked a regular cigarette**	Less than 1 hour ago	1-6 hours ago	7-12 hours ago	12-24 hours ago	1-7 days ago	Not at all in last 7 days
**Smoked any other tobacco** (cigar, cigarillo, bidi, shisha, etc.)	Less than 1 hour ago	1-6 hours ago	7-12 hours ago	12-24 hours ago	1-7 days ago	Not at all in last 7 days
**Smoked cannabis/marijuana**	Less than 1 hour ago	1-6 hours ago	7-12 hours ago	12-24 hours ago	1-7 days ago	Not at all in last 7 days
**Vaped cannabis/marijuana**	Less than 1 hour ago	1-6 hours ago	7-12 hours ago	12-24 hours ago	1-7 days ago	Not at all in last 7 days
**Used smokeless tobacco** (chew, pinch, snuff, snus)	Less than 1 hour ago	1-6 hours ago	7-12 hours ago	12-24 hours ago	1-7 days ago	Not at all in last 7 days
**Used nicotine replacement therapy** (patches, gum, lozenges, etc.) **or nicotine pouches**	Less than 1 hour ago	1-6 hours ago	7-12 hours ago	12-24 hours ago	1-7 days ago	Not at all in last 7 days
**Ate grilled meat** (i.e, cooked over flame or charcoal, or with black grill marks)	Less than 1 hour ago	1-6 hours ago	7-12 hours ago	12-24 hours ago	1-7 days ago	Not at all in last 7 days
**Were in the presence of someone smoking cigarettes or tobacco inside** (home, car, etc.)	Less than 1 hour ago	1-6 hours ago	7-12 hours ago	12-24 hours ago	1-7 days ago	Not at all in last 7 days

Please tell us more about the LAST e-cigarette you used *(if applicable):*

1. **What brand was the device?** (*please be as specific as possible*) ________________________________

2. **What brand was the cartridge, pod or e-liquid?** (*please be as specific as possible*) ________________________________

3. **What flavour was the cartridge, pod or e-liquid?** ________________________________

4. a) **Did it contain nicotine?** (*circle one*)
   Yes →
   No
   Don't know

   b) **What concerntration of nicotine was it?** (*answer in mg/ml OR %*)
   _________mg/ml **OR** _______% nicotine
   Don't know

   c) **Was it nicotine salt?** (*circle one*)
   Yes
   No
   Don't know

PLEASE INCLUDE THIS FORM WITH YOUR URINE SAMPLES IN THE ENVELOPE PROVIDED.

WAVE 4.5 – UPDATES

IMPORTANT: PLEASE FILL THIS OUT IMMEDIATELY AFTER COLLECTING YOUR URINE SAMPLE.

Note: We will NOT test your urine for cannabis or any illegal substances. We only ask about cannabis (and grilled meat) below because they can produce chemicals in your urine similar to smoking and vaping.

DATE OF COLLECTION: _______________ TIME OF COLLECTION: _______________am / pm

When was the LAST time you did the following…

Please circle one response per row. If you have never done the following, select 'Not at all in last 7 days'.

**Used an e-cigarette/vaped**	Less than 1 hour ago	1-6 hours ago	7-12 hours ago	12-24 hours ago	1-7 days ago	Not at all in last 7 days
**Smoked a regular cigarette**	Less than 1 hour ago	1-6 hours ago	7-12 hours ago	12-24 hours ago	1-7 days ago	Not at all in last 7 days
**Smoked any other tobacco** (cigar, cigarillo, bidi, shisha, etc.)	Less than 1 hour ago	1-6 hours ago	7-12 hours ago	12-24 hours ago	1-7 days ago	Not at all in last 7 days
**Smoked cannabis/marijuana**	Less than 1 hour ago	1-6 hours ago	7-12 hours ago	12-24 hours ago	1-7 days ago	Not at all in last 7 days
**Vaped cannabis/marijuana**	Less than 1 hour ago	1-6 hours ago	7-12 hours ago	12-24 hours ago	1-7 days ago	Not at all in last 7 days
**Used smokeless tobacco** (chew, pinch, snuff, snus)	Less than 1 hour ago	1-6 hours ago	7-12 hours ago	12-24 hours ago	1-7 days ago	Not at all in last 7 days
**Used nicotine replacement therapy** (patches, gum, lozenges, etc.) **or nicotine pouches**	Less than 1 hour ago	1-6 hours ago	7-12 hours ago	12-24 hours ago	1-7 days ago	Not at all in last 7 days
**Ate grilled meat** (i.e, cooked over flame or charcoal, or with black grill marks)	Less than 1 hour ago	1-6 hours ago	7-12 hours ago	12-24 hours ago	1-7 days ago	Not at all in last 7 days
**Were in the presence of someone smoking cigarettes or tobacco inside** (home, car, etc.)	Less than 1 hour ago	1-6 hours ago	7-12 hours ago	12-24 hours ago	1-7 days ago	Not at all in last 7 days

Please tell us more about the LAST e-cigarette you used *(if applicable):*

1. **What brand was the device?**
   (*please be as specific as possible*) ____
2. **What brand was the cartridge, pod or e-liquid?**
   (*please be as specific as possible*) ____
3. **What flavour was the cartridge, pod or e-liquid?** ____
4. a) **Did it contain nicotine?** (*circle one*)
   Yes →
   No
   Don't know

   b) **What concentration of nicotine was it?** (*answer in mg/ml OR %*)
   _________mg/ml **OR** _______% nicotine
   Don't know

   c) **Was it nicotine salt?** (*circle one*)
   Yes
   No
   Don't know

PLEASE INCLUDE THIS FORM WITH YOUR URINE SAMPLES IN THE ENVELOPE PROVIDED.
