# Supplementary material for: Nicotine Exposure From Smoking Tobacco and Vaping Among Adolescents
Source: JAMA Netw Open. 2025 Mar 12;8(3):e2462544. doi: 10.1001/jamanetworkopen.2024.62544 (PMC11904731; doi:10.1001/jamanetworkopen.2024.62544)
Supplement: Supplement 2. — Data Sharing Statement [file jamanetwopen-e2462544-s002.pdf]

Data Sharing Statement

Hammond. Nicotine Exposure From Smoking Tobacco and Vaping Among Adolescents. *JAMA Netw Open*. Published March 12, 2025. doi:10.1001/jamanetworkopen.2024.62544

Data
Data available: Yes
Data types: Deidentified participant data, Data dictionary
How to access data: Deidentified study data may be made available on request to researchers who submit a proposal that is approved by the principal investigator. Proposals should be submitted to David Hammond (dhammond@uwaterloo.ca).
When available: With publication

Supporting Documents
Document types: None

Additional Information
Who can access the data: Deidentified study data may be made available on request to researchers who submit a proposal that is approved by the principal investigator.
Types of analyses: Data will be made available for the specific purposes approved in the proposal.
Mechanisms of data availability: Data will be made available after approval of a proposal and with a signed data access and use agreement.
